# Specific ligands of the peripheral benzodiazepine receptor induce apoptosis and cell cycle arrest in human colorectal cancer cells

**DOI:** 10.1054/bjoc.2001.2181

**Published:** 2001-11

**Authors:** K Maaser, M Höpfner, A Jansen, G Weisinger, M Gavish, A P Kozikowski, A Weizman, P Carayon, E-O Riecken, M Zeitz, H Scherübl

**Affiliations:** Medical Clinic I, Gastroenterology/Infectious Diseases/Rheumatology, Benjamin Franklin University Hospital, Free University of Berlin, Germany; Department of Endocrinology, Sackler Faculty of Medicine, Tel Aviv University, Israel; Department of Pharmacology, The Bruce Rappaport Faculty of Medicine, Technion-Israel Institute of Technology, Haifa, Israel; Drug Discovery Program, Georgetown University Medical Center, Washington, USA; Research Unit, Geha Psychiatric Hospital and Laboratory of Biological Psychiatry, Felsenstein Medical Research Center, Beilinson Medical Center, Petah Tiqva and Sackler Faculty of Medicine, Tel Aviv, Israel; Department of Immunology-Oncology, Sanofi-Synthelabo Research, Montpellier, 34184, France

**Keywords:** peripheral benzodiazepine receptor, colorectal cancer, cell proliferation, apoptosis, cell cycle

## Abstract

The peripheral benzodiazepine receptor (PBR) has been implicated in growth control of various tumour models. Although colorectal cancers were found to overexpress PBR, the functional role of PBR in colorectal cancer growth has not been addressed to date. Using primary cell cultures of human colorectal cancers and the human colorectal carcinoma cell lines HT29, LS174T, and Colo320 DM we studied the involvement of PBR in the growth control and apoptosis of colorectal cancers. Both mRNA and protein expression of PBR were detected by RT-PCR and flow cytometry. Using confocal laser scanning microscopy and immunohistochemistry the PBR was localized in the mitochondria. The specific PBR ligands FGIN-1-27, PK 11195, or Ro5-4864 inhibited cell proliferation dose-dependently. FGIN-1-27 decreased the mitochondrial membrane potential, which indicates an early event in apoptosis. Furthermore, FGIN-1-27, PK 11195 or Ro5-4864 increased caspase-3 activity. In addition to their apoptosis-inducing effects, PBR ligands induced cell cycle arrest in the G _1_/G _0_-phase. Thus, our data demonstrate a functional involvement of PBR in colorectal cancer growth and qualify the PBR as a possible target for innovative therapeutic approaches in colorectal cancer. © 2001 Cancer Research Campaign http://www.bjcancer.com


*British Journal of Cancer* (2001) **85**(11), 1771–1780
© 2001 Cancer Research Campaign
doi: 10.1054/ bjoc.2001.2181, available online at http://www.idealibrary.com on IDEAL

### Specific ligands of the peripheral benzodiazepine receptor induce apoptosis and cell cycle arrest in human colorectal cancer cells

**K Maaser[1], M Höpfner[1], A Jansen[1], G Weisinger[2], M Gavish[3], AP Kozikowski[4], A Weizman[5], P Carayon[6], E-O Riecken[1], M Zeitz[1] and H Scherübl[1]**

[1]Medical Clinic I, Gastroenterology/Infectious Diseases/Rheumatology, Benjamin Franklin University Hospital, Free University of Berlin, Germany; [2]Department of Endocrinology, Sackler Faculty of Medicine, Tel Aviv University, Israel; [3]Department of Pharmacology, The Bruce Rappaport Faculty of Medicine, Technion-Israel Institute of Technology, Haifa, Israel; [4]Georgetown University Medical Center, Drug Discovery Program, Washington, USA; [5]Research Unit, Geha Psychiatric Hospital and Laboratory of Biological Psychiatry, Felsenstein Medical Research Center, Beilinson Medical Center, Petah Tiqva, and Sackler Faculty of Medicine, Tel Aviv, Israel; [6]Department of Immunology-Oncology, Sanofi-Synthelabo Research, 34184 Montpellier, France

**Summary** The peripheral benzodiazepine receptor (PBR) has been implicated in growth control of various tumour models. Although colorectal cancers were found to overexpress PBR, the functional role of PBR in colorectal cancer growth has not been addressed to date. Using primary cell cultures of human colorectal cancers and the human colorectal carcinoma cell lines HT29, LS174T, and Colo320 DM we studied the involvement of PBR in the growth control and apoptosis of colorectal cancers. Both mRNA and protein expression of PBR were detected by RT-PCR and flow cytometry. Using confocal laser scanning microscopy and immunohistochemistry the PBR was localized in the mitochondria. The specific PBR ligands FGIN-1-27, PK 11195, or Ro5-4864 inhibited cell proliferation dose-dependently. FGIN-1-27 decreased the mitochondrial membrane potential, which indicates an early event in apoptosis. Furthermore, FGIN-1-27, PK 11195 or Ro5-4864 increased caspase-3 activity. In addition to their apoptosis-inducing effects, PBR ligands induced cell cycle arrest in the $G_1/G_0$-phase. Thus, our data demonstrate a functional involvement of PBR in colorectal cancer growth and qualify the PBR as a possible target for innovative therapeutic approaches in colorectal cancer. © 2001 Cancer Research Campaign http://www.bjcancer.com

**Keywords**: peripheral benzodiazepine receptor; colorectal cancer; cell proliferation; apoptosis; cell cycle

Benzodiazepines bind not only to $GABA_A$-receptors, but also to the structurally and pharmacologically distinct peripheral benzodiazepine receptor (PBR) (Braestrup and Squires, 1977). In contrast to $GABA_A$-receptors, the PBR exhibits high affinity for the benzodiazepine Ro5-4864, the isoquinoline carboxamide PK 11195 and the indoleacetamide FGIN-1-27 (Le Fur et al, 1983; Kozikowski et al, 1993), but only a very low affinity for the benzodiazepine clonazepam (Wang et al, 1984a). PBR is highly expressed in steroid-producing tissues such as ovary, testis, adrenal and placenta (Gavish et al, 1999; Beurdeley-Thomas et al, 2000). In contrast, PBR is present only at low densities in skeletal muscle, the gastrointestinal tract, and in much of the brain (Verma and Snyder, 1989; Gavish et al, 1992). Interestingly, PBR was shown to be overexpressed in several tumours including those of the colon, brain, breast, ovary and liver (Katz et al, 1990a, 1990b; Cornu et al, 1992; Batra and Iosif, 1998; Venturini et al, 1998; Carmel et al, 1999; Hardwick et al, 1999). PBR is mainly localized in the mitochondria (Anholt et al, 1986), but has also been detected in the plasma membrane (Garnier et al, 1993) and nucleus (Hardwick et al, 1999). Due to the pharmacological effects of specific PBR ligands and the cellular and subcellular localization of PBR, a broad spectrum of putative functions has been attributed to PBR such as regulation of steroid production (Papadopoulos, 1993), inflammatory response (Torres et al, 1999), insulin secretion (Marchetti et al, 1996b), mitochondrial respiration (Hirsch et al, 1989; Krueger, 1995), cell differentiation (Canat et al, 1993) and cell proliferation. Proliferation of various tumours including breast cancer (Beinlich et al, 1999; Carmel et al, 1999), melanoma (Landau et al, 1998), testis (Garnier et al, 1993) and astrocytoma (Neary et al, 1995) was shown to be inhibited by PBR ligands at micromolar concentrations. In contrast, at nanomolar concentrations PBR-specific ligands were mitogenic (Laird et al, 1989; Ikezaki and Black, 1990; Beinlich et al, 1999; Hardwick et al, 1999). However, the underlying mechanisms of the growth-modulating actions of PBR ligands are still unknown.

Tumour cell growth is characterized by an imbalance between cell division and cell death. A common step in tumorigenesis is the loss of the capability of cells to undergo apoptosis. The localization of PBR in the mitochondrial membrane and its implication in the permeability transition pore (Zorov, 1996; Fennell et al, 2001) suggest that the PBR takes part in the regulation of the mitochondrial permeability and induction of apoptosis. However, the role of PBR in the regulation of apoptosis is not yet understood. Specific PBR ligands were shown to either induce apoptosis directly (Marchetti et al, 1996a; Tanimoto et al, 1999; Fischer et al, 2001), or to facilitate apoptosis by inhibiting the anti-apoptotic effects of BCL-2 (Hirsch et al, 1998; Larochette et al, 1999; Ravagnan et al, 1999). In contrast, apoptosis-protective effects of PBR ligands have been reported as well (Bono et al, 1999). Besides their putative role in the regulation of apoptosis,

*Received 5 March 2001*
*Revised 10 August 2001*
*Accepted 10 August 2001*

*Correspondence to:* H Scherübl

PBR ligands were shown to induce cell cycle arrest in the $G_1/G_0$- and $G_2$/M-phase in breast carcinoma and melanoma cell lines (Landau et al, 1998; Carmel et al, 1999). Similarly, the new benzazepine BBL22, classified as a PBR-specific ligand, induced arrest in the $G_2$/M-phase in different tumour cell lines of epithelial or haematopoietic origin followed by an induction of apoptosis (Xia et al, 2000).

As PBR was shown to be overexpressed in colorectal tumours (Katz et al, 1990b), PBR may well play a functional role in colorectal carcinogenesis. However, an involvement of PBR in colorectal cancer growth has not been investigated so far. In this study we show that specific PBR ligands inhibit proliferation of colorectal cancer cells, which is associated with an induction of apoptosis and cell cycle arrest.

### MATERIALS AND METHODS

### Cell culture

The human colorectal adenocarcinoma cell lines HT29 and Colo320 DM (neurorendocrine-differentiated) were grown in RPMI 1640 medium supplemented with 10% fetal calf serum. The human colorectal adenocarcinoma cell line LS174T was grown in Dulbecco's minimal essential medium supplemented with 10% fetal calf serum. Cell lines were cultured in a humidified atmosphere containing 5% $CO_2$ at 37˚C (Hanski et al, 2000).

Surgically resected specimens of primary colorectal carcinomas (3 rectum, 3 colon) were obtained from 4 female and 2 male patients who underwent surgery in the Department of Surgery, Benjamin Franklin University Hospital, Free University Berlin. The human tumour material was used according to the standards set by the Ethical Committee of the Benjamin Franklin University Hospital, Free University of Berlin. The age of the patients ranged from 23 to 78 years. Primary cell cultures were prepared by mechanical dissection using a Medimachine with 50 µm Medicons (Becton Dickinson, Heidelberg, Germany) according to the manufacturer's instructions. Isolated cells were cultured in RPMI 1640 medium supplemented with 10% fetal calf serum, penicillin (100 U $ml^{-1}$), and streptomycin (100 µg $ml^{-1}$) and cultured in a humidified atmosphere containing 5% $CO_2$ at 37˚C (Höpfner et al, 1998).

### Reverse transcriptase chain reaction (RT-PCR)

RNA isolation, reverse transcription, and PCR reactions were carried out as described (Glassmeier et al, 1998). Amplification consisted of 38 cycles with the following conditions for denaturation, annealing and extension: 94˚C for 45 s, 60˚C for 45 s and 72˚C for 2 min. The primers for amplification of cDNA were designed using the PRIMER program (Whitehead Institute for Biomedical Research, Cambridge, MA, USA) based on the cDNA sequences of human 18 kDa PBR subunit obtained from the GenBank (accession number: M36035). Primers for amplification were:

Forward: 5′-CACGCTCTACTCAGCCATGG-3′
Reverse: 5′-GCAGTAGTTGAGTGTGGTCGC-3′

The expected PCR product size was 298 bp. PCR products were sequenced on an ABI 310 sequencer (Applied Biosystems, Foster City, CA, USA) and specificity of the transcripts was confirmed by the NCBI (National Center for Biotechnology Information, Bethesda, MD, USA) BLASTIN 2.0 search program (Altschul et al, 1997).

### Flow cytometry

Cells were trypsinized, washed twice with phosphate-buffered NaCl solution (PBS; 140 mM NaCl, 10 mM $Na_2HPO_4$, 2.6 mM KCl, 1.4 mM $KH_2PO_4$, pH 7.4) and immunoassayed, as described (Maaser et al, 1999; Stoebner et al, 1999). Cells were fixed in 4% paraformaldehyde for 30 min at room temperature, washed once with PBS, and permeabilized for 10 min in PBS containing 0.1% saponin (Merck, Darmstadt, Germany) and 1% bovine serum albumin (BSA; Sigma, Deisenhofen, Germany). Cells were incubated for 1 h at room temperature with primary anti-PBR antibody 8D7 (5 µg $ml^{-1}$ in PBS containing 0.1% saponin, 1% BSA) (Dussossoy et al, 1996) or isotypic control mouse IgG1 (DAKO, Hamburg, Germany), respectively. Cells were washed twice with PBS containing 0.03% saponin and then incubated with 4 µg $ml^{-1}$ secondary Alexa™ 488-labelled goat-anti-mouse IgG antibody (Molecular Probes, Eugene, OR, USA) for 1 h at room temperature. Fluorescence was detected by flow cytometry on a FACSCalibur (Becton Dickinson, Heidelberg, Germany) and analysed using CellQuest software.

### Immunofluorescence labelling and confocal microscopy

Cells were plated on glass cover slips for 24 h. Cells were stained with the mitochondrial dye CMTMRos (100 nM in medium, 30 min, 37˚C) (Molecular Probes, Eugene, OR, USA). After washing with PBS, the samples were fixed by incubation with 4% paraformaldehyde in PBS for 30 min at room temperature, washed with PBS, and permeabilized with cold methanol (20 min, –20˚C) followed by acetone (10 s, –20˚C). Cells were incubated for 1 h at room temperature with primary anti-PBR monoclonal antibody 8D7 (5 µg $ml^{-1}$ in PBS) (Dussossoy et al, 1996) or isotypic control mouse IgG1 (DAKO, Hamburg, Germany), respectively. Thereafter, cells were washed with PBS twice and incubated with 4 µg $ml^{-1}$ secondary Alexa™ 488-labelled goat-anti-mouse IgG antibody (Molecular Probes, Eugene, OR, USA) for 1 h at room temperature. Fluorescence and transmission images were obtained using the inverted confocal microscope LSM 410 with a 63x /1,2 W Korr objective (Zeiss, Oberkochen, Germany).

### Immunohistochemistry

23 paraffin-embedded colorectal carcinomas were studied. These were 1 well differentiated, 13 moderately differentiated, and 9 poorly differentiated cancers. 2 µm sections of the tumours were deparaffinized and rehydrated in a series of alcohol solutions of decreasing concentrations (Grabowski et al, 2000, 2001). Then sections were transferred into a robotic machine (Chemo-mate, DAKO, Heidelberg, Germany) and staining procedure was automatically performed under following standard conditions. Sections were incubated with the anti-PBR antibody 8D7 (0.5 µg $ml^{-1}$) for 30 min at room temperature. After washing samples were incubated with the second anti-mouse IgG (1:20 dilution) (DAKO, Heidelberg, Germany) for 30 min at room temperature. The APAAP complex (DAKO, Heidelberg, Germany) was incubated for 30 min at a dilution 1:50 in RPMI medium containing 10% fetal calf serum and 1% sodium azide. Staining was detected using fast-red system (DAKO, Heidelberg, Germany) and samples were counterstained with haemalaun.

### Cell proliferation assay

The ability of the PBR ligands to modulate cell proliferation was studied using the crystal violet method (Gillies et al, 1986). In

 © 2001 Cancer Research Campaign brief, cells were seeded on 96-well plates at a density of 1000 cells well$^{-1}$ (HT29 and Colo320 DM) or 2000 cells well$^{-1}$ (LS174T). After 72 h the PBR ligands FGIN-1-27, PK 11195 (Tocris, Bristol, UK), or Ro5-4685 (Sigma, Deisenhofen, Germany) were added at concentrations of 1 nM to 100 μM. The FGIN–1-52 (Kozikowski et al, 1993) or the $GABA_A$ receptor ligand clonazepam (Sigma, Deisenhofen, Germany) were used as controls. Each concentration group consisted of 10 wells. The incubation medium was changed every day. Cell quantification was performed after 0, 24, 48, 72 and 96 h of incubation. Cells of each well were washed (200 μl PBS) and fixed (100 μl, 1% glutaraldehyde in PBS for 15 min at room temperature). After another washing step (200 μl PBS), cells were stained with 0.1% crystal violet in PBS for 30 min at room temperature. The unbound dye was removed by washing with $H_2O$ for 30 min. Crystal violet which had absorbed onto the cells was solubilized with 100 μl 0.2% Triton-X–100 in PBS for at least 24 h at 37˚C. The crystal violet containing solution was measured spectrometrically at 570 nm using an ELISA-Reader. In the range of 500 to 20 000 cells well$^{-1}$, the measured extinction was a linear function of the cell number (Höpfner et al, 1998).

### Mitochondrial membrane potential measurement

Cells were incubated with medium containing FGIN-1-27, PK 11195, Ro5-4685, FGIN–1-52, or clonazepam at concentrations from 10 μM to 100 μM for 6 h to 16 h. Changes in mitochondrial membrane potential ($\Delta\Psi_M$) were assessed using the fluorogenic dye JC–1 (Molecular Probes, Eugene, OR, USA). The lipophilic cationic dye JC–1 stained mitochondrial membranes independent of the potential, thereby emitting green fluorescence (530 nm) reflecting the mitochondrial volume (Mancini et al, 1997). Upon potential-dependent accumulation within the mitochondria JC–1 forms aggregates emitting light at 590 nm. $\Delta\Psi_M$ is indicated by 590/530 nm JC–1 emission ratio (Cossarizza et al, 1993). Cells were stained with 1 ml JC–1 (1 μg ml$^{-1}$, 15 min, 37˚C) and subsequently washed twice with ice-cold PBS. Fluorescence was analysed by flow cytometry. To confirm the data obtained by JC–1 staining, a second mitochondrial dye CMTMRos (Molecular Probes, Eugene, OR, USA) was used (100 nM, 30 min, 37˚C). The fluorescence emission of CMTMRos stained cells at 590 nm directly correlates with the $\Delta\Psi_M$ (Petit et al, 1990).

### Caspase-3 activity assay

Cells were incubated with medium containing FGIN-1-27, PK 11195, Ro5-4685, FGIN–1-52, or clonazepam at concentrations from 10 μM to 100 μM for 6 h to 16 h, washed twice with PBS, and stored at –80˚C until use. Approximately $10^6$ cells were lysed with 500 μl lysis buffer (10 mM Tris-HCl, 10 mM $NaH_2PO_4/Na_2HPO_4$, 130 mM NaCl, 1% Triton-X–100, 10 mM $NaPP_i$, pH 7.5) and total protein content was quantified using the BCA protein assay kit (Pierce, Rockford, IL, USA). The activity of caspase-3 was calculated from the cleavage of the fluorogenic substrate DEVD-AMC (Nicholson et al, 1995). In brief, 100 μl cell lysate containing 500 μg ml$^{-1}$ protein was incubated with 100 μl substrate solution (2 μg caspase-3 substrate AC-DEVD-AMC, 20 mM HEPES, 10% glycerol, 2 mM DTT, pH 7.5) for 1 h at 37˚C. The cleavage of DEVD-AMC was measured with a VersaFluor fluorometer (Biorad, Munich, Germany) using a 360 nm excitation and a 460 nm emission wavelength.

### Cell cycle analysis

Cell cycle analysis was performed by the method of Vindelov and Christensen (Vindelov and Christensen, 1990). $5 \times 10^5$ cells per well were cultured for 24 h and then exposed to PBR ligands for another 24 h. Cells were trypsinized, washed and the nuclei were isolated using CycleTest PLUS DNA Reagent Kit (Becton Dickinson, Heidelberg, Germany). According to the manufacturer's instructions DNA was stained with propidium iodide. The DNA content of the nuclei was detected by flow cytometry and analysed using CellFit software (Becton Dickinson, Heidelberg, Germany).

### Statistical analysis

Comparison of multiple means was performed with nonparametric ANOVA. Comparison of individual drug treatments to control treatments was performed with the unpaired, 2-tailed Mann–Whitney U test for proliferation, $\Delta\Psi_M$ measurements, and caspase-3 activity experiments. Data are expressed as mean percentage of control ± SEM. For cell cycle analysis the unpaired student's *t*-test was used. *P* values were considered to be significant at < 0.05.

### RESULTS

### PBR expression in colorectal cancer cells

The mRNA expression of PBR was investigated by RT-PCR. Both in HT29 cells and in primary cultures of colorectal carcinomas, PBR mRNA was detected (Figure 1A). The specificity of the obtained PCR products was confirmed by direct sequencing of cDNAs. HT29 cells did not express any β-chain of the $GABA_A$-receptor (data not shown), which are essential for benzodiazepines binding to the $GABA_A$-receptor. To investigate the expression of the PBR protein, cells were stained with the monoclonal anti-PBR antibody 8D7 and fluorescence was analysed by flow cytometry. The expression of PBR protein was detected in primary cell cultures of colorectal cancer cells (Figure 1B), as well as in the cell lines HT29, LS174T and Colo320 DM (Figure 1C–E). In non-permeabilized cells, no specific 8D7 fluorescence was observed indicating an intracellular localization of PBR (Figure 1C–E).

### PBR localization in mitochondria

To further characterize the subcellular localization of PBR, HT29 cells were simultaneously stained with the mitochondrial dye CMTMRos and the anti-PBR antibody 8D7. Confocal laser scanning microscopy imaged the subcellular distribution of fluorescence. Green fluorescence of 8D7-labelled PBR was detected within the cytoplasm but neither in the cell membrane nor in the nucleus (Figure 2A, E). The staining pattern was comparable to the one obtained by the mitochondrial dye CMTMRos (Figure 2B, I). Superposition of the images of 8D7-labelled PBR and CMTMRos stained mitochondria resulted in a yellow colour (Figure 2C) indicating a co-localization of PBR and mitochondria. Likewise, PBR was localized in the mitochondria in the colorectal cell lines LS174T and Colo320 DM as well as in primary cultures of 6 resected colorectal cancers (data not shown). To further verify the mitochondrial localization of PBR, subcellular PBR expression was immunohistochemically determined in 23 other colorectal cancers. The differentiation grade of these tumours ranged from well (*n* = 1), moderately (*n* = 13), to poorly (*n* = 9) differentiated.

© 2001 Cancer Research Campaign

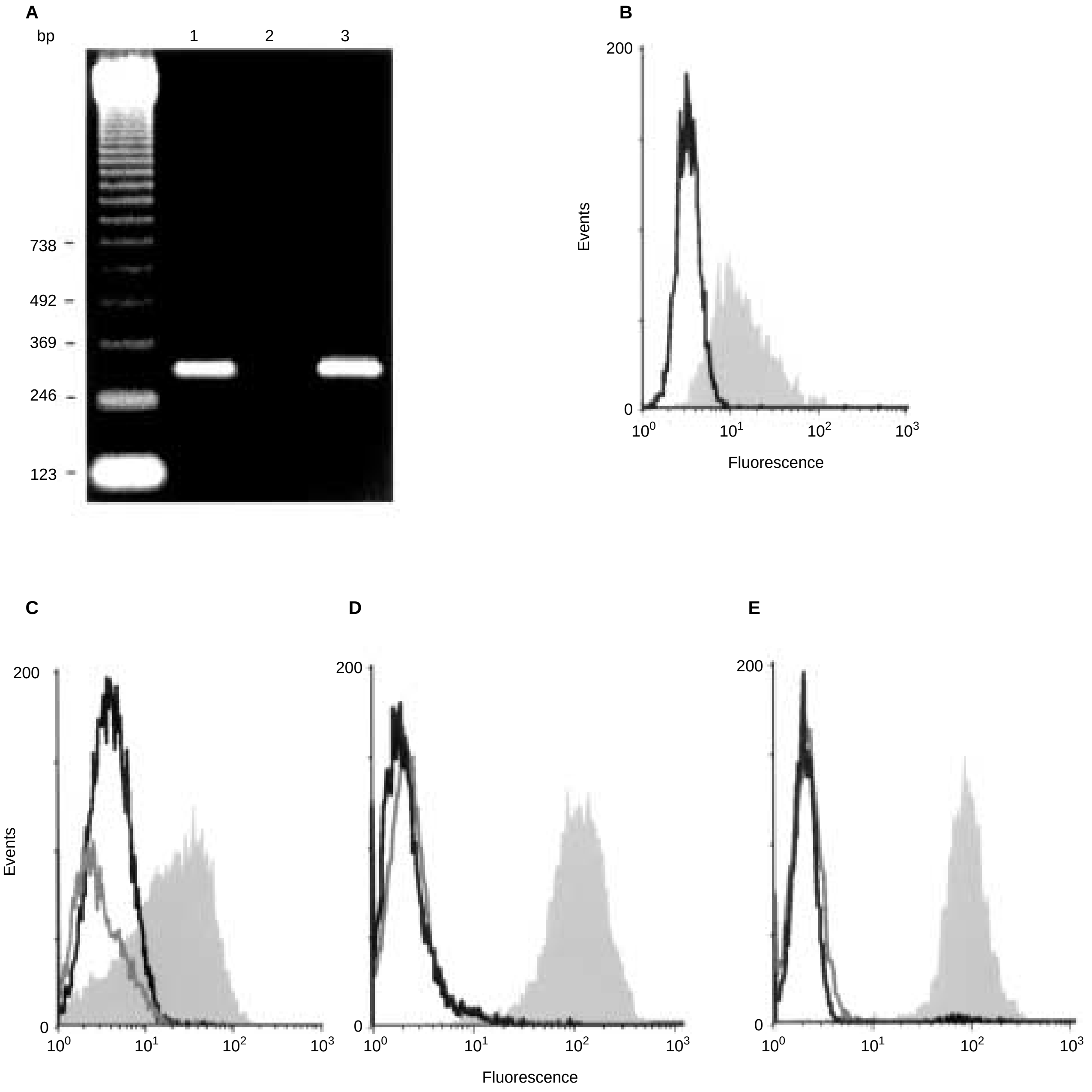

**Figure 1** PBR mRNA and protein expression. (**A**) PBR mRNA expression in HT29 cells (lane 1) and in primary cell cultures of human colorectal cancers (lane 3) was detected by RT-PCR. Negative control was performed by omitting reverse transcriptase (lane 2). (**B**–**E**) Flow cytometric analysis of PBR expression in a primary cell culture of a colorectal cancer (**B**), and in the colorectal cell lines HT29 (**C**), LS174T (**D**), and Colo320 DM (**E**). Cells were stained with the specific PBR antibody 8D7 with previous membrane permeabilization (grey area) or without previous membrane permeabilization (grey line, **C**–**E**). Black line: isotypic control

In all 23 cancers, the specific PBR staining was observed unevenly distributed within the cytoplasm (Figure 2K, L). No specific PBR staining was observed in the plasma membrane nor in the nuclei of cells, indicating that in colorectal cancers PBR is located in the mitochondria.

### Inhibition of cell proliferation by specific PBR ligands

Growth-modulating effects of PBR ligands were studied by the crystal violet assay. The specific PBR ligands FGIN-1-27, PK 11195 and Ro5-4864 (10 μM to 100 μM) significantly inhibited the proliferation of HT29 cells in a dose-dependent manner (Figure 3). After 72 h of incubation FGIN-1-27 decreased cell growth with an $IC_{50}$ = 14 ± 2 μM, thus showing greater efficacy than PK 11195 ($IC_{50}$ = 58 ± 9 μM) and Ro5-4864 ($IC_{50}$ = 83 ± 13 μM). At lower concentrations (1 nM–1 μM) of FGIN-1-27, PK 11195 or Ro5-4864, no proliferation-modulating effects were detected, regardless of the presence or absence of serum in the medium (data not shown). The benzodiazepine clonazepam and the indoleacetamide FGIN–1-52 were used as control agents. Despite the similarity of their chemical structures to Ro5-4864 or FGIN-1-27 (Table 1), respectively, both agents displayed almost no affinity to PBR (Wang et al, 1984a; Kozikowski et al, 1993). In contrast to the antiproliferative action of the specific PBR ligands,

 © 2001 Cancer Research Campaign

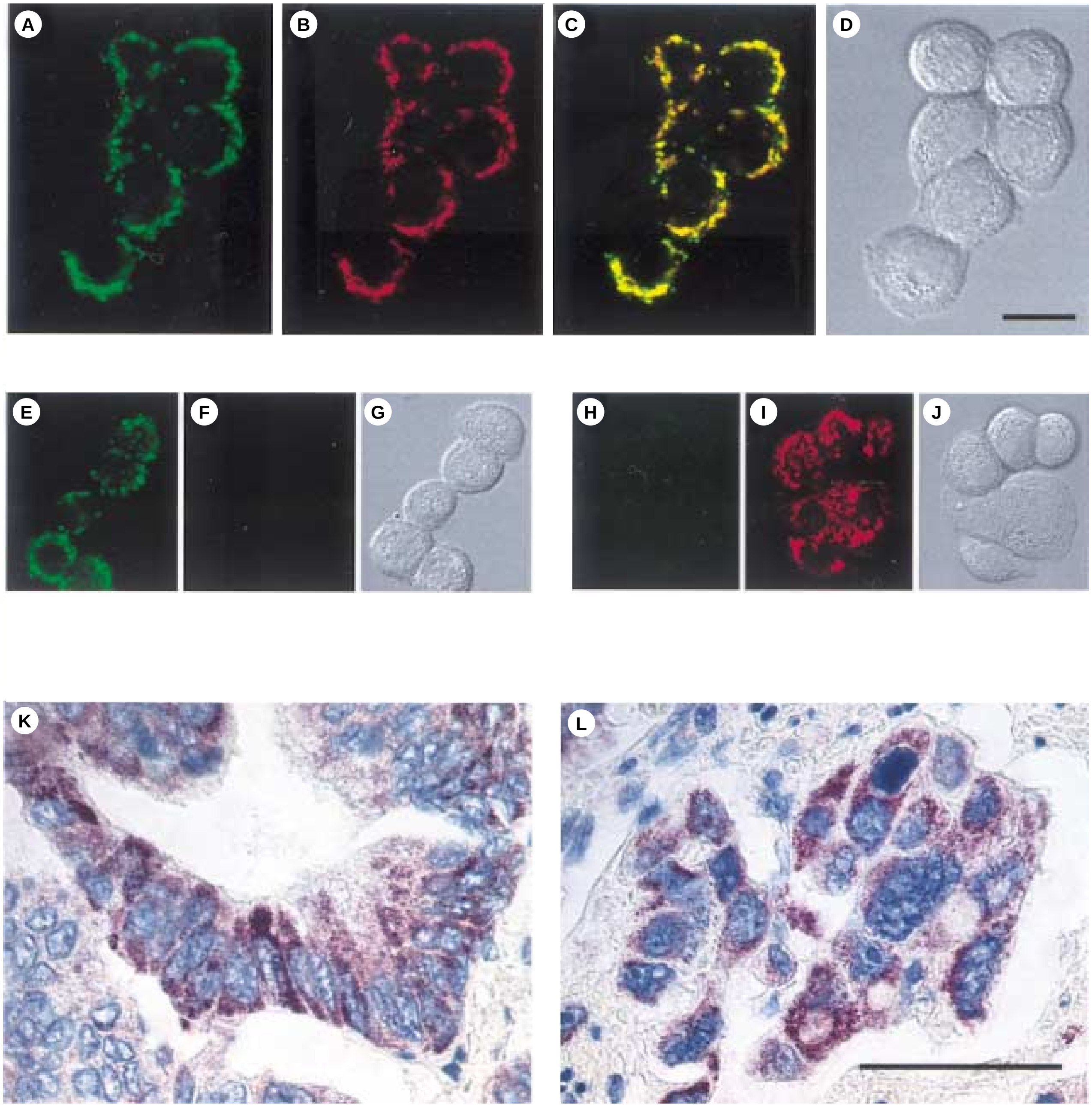

**Figure 2** PBR localization in the mitochondria of HT29 cells. (**A**–**J**) Confocal laser scanning microscopy simultaneously visualized PBR and mitochondria. PBR was immunoassayed with the specific monoclonal antibody 8D7 (**A**, **E**), and mitochondria were marked with CMTMRos (**B**, **I**). Superposition of both fluorescence images resulted in a bright yellow colour (**C**), indicating a co-localization of PBR and mitochondria. Specificity of imaging was shown by omitting CMTMRos staining (**F**) or using isotypic control antibody (**H**), respectively. Corresponding transmission light images are shown (**D**, **G**, **J**). Bar = 10 µM. (**K**, **L**) Sections of paraffin-embedded tumours were immunohistochemically stained with anti-PBR antibody 8D7 and PBR expression was detected using the APAAP system (red colour). (**K**) Colorectal carcinoma classified as moderately differentiated. (**L**) Colorectal carcinoma classified as poorly differentiated. Bar = 100 µm clonazepam or FGIN–1-52 caused little if any reduction in proliferation (Figure 3), indicating that the growth-inhibiting effects are PBR-specific. Likewise to HT29 cells, the specific PBR ligands inhibited the proliferation of the colorectal cancer cell lines LS174T and Colo320 DM. After 72 h of incubation FGIN-1-27 decreased the growth of LS174T or Colo320 DM cells with $IC_{50}$ of 16 ± 2 µM or of 19 ± 1 µM, respectively. PK11195 inhibited cell proliferation with $IC_{50}$ = 49 ± 4 µM (LS174T) and $IC_{50}$ = 53 ± 4 µM (Colo320 DM), respectively.

### Induction of mitochondrial alterations by specific PBR ligands

PBR is located mainly in the outer mitochondrial membrane and is thought to form the permeability transition pore, which plays an important role in the regulation of $\Delta\psi_M$. Therefore, we investigated if specific PBR ligands can modulate the $\Delta\psi_M$ and mitochondrial volume. FGIN-1-27 significantly depolarized the mitochondria of HT29 cells in a dose-dependent manner as assayed by the

© 2001 Cancer Research Campaign

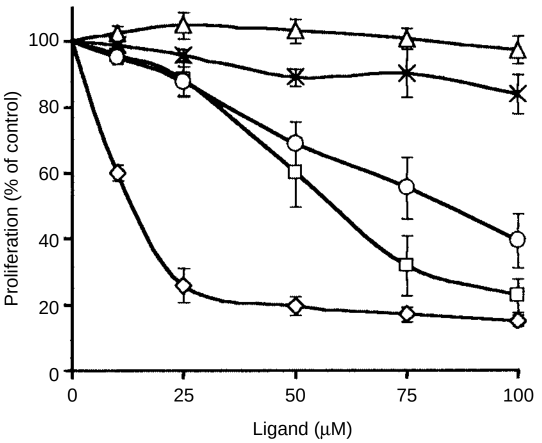

**Figure 3** Antiproliferative effects of PBR ligands in HT29 cells. The specific PBR ligands FGIN-1-27 (◇), PK 11195 (□), and Ro5-4864 (○) dose-dependently inhibited proliferation of HT29 cells as shown for a 72 h incubation period. Growth inhibition versus control was significant for FGIN-1-27 and Ro5-4864 from 10 μM to 100 μM, and for PK 11195 from 25 μM to 100 μM ($P < 0.05$). In contrast, the $GABA_A$-receptor ligand clonazepam (Δ) or the 'no-affinity' ligand FGIN-1-52 (×) showed no or only minor effects. Means of 4 to 6 independent experiments ± SEM are shown

**Table 1** Structures and $K_i$ values of FGIN-1-27 and FGIN-1-52. $K_i$ values indicate the displacement of [$^3$H]4′-chlorodiazepam from rat cerebellar glial cell membranes (Kozikowski et al, 1993)

| FGIN-1-27 | FGIN-1-52 |
|---|---|
|  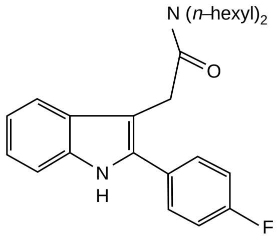 |  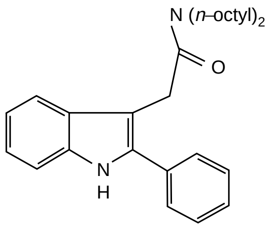 |
| $K_i$ = 4.4 ± 0.1 nM | $K_i$ > 1000 nM |

mitochondrial dye JC–1. Likewise, $\Delta\psi_M$ of primary cell cultures of colorectal cancers was significantly decreased by 50 μM FGIN-1-27 (Figure 4A). The results were confirmed by using the mitochondrial dye CMTMRos. CMTMRos stained HT29 cells displayed mean fluorescence of 83 ± 6% (10 μM), 68 ± 4% (50 μM), and 64 ± 6% (100 μM) of control, respectively, when incubated for 6 h with FGIN-1-27. Simultaneously, an increase of mitochondrial volume of HT29 cells and primary cultures was detected (Figure 4A), indicating that FGIN-1-27 not only induced a mitochondrial membrane depolarization but also an increase in mitochondrial volume. In contrast to FGIN-1-27, PK 11195 or Ro5-4864 showed no effects on HT29 cells after 1 h to 16 h of incubation. Moreover, the non-PBR-specific compound FGIN–1-52 did not alter the $\Delta\psi_M$ (data not shown). In control experiments the $K^+$ ionophore valinomycin was used to reduce the JC–1 590 nm fluorescence (Cossarizza et al, 1993) resulting in a mean 590 nm fluorescence of one third of corresponding control.

### Induction of apoptosis by specific PBR ligands

To date, an involvement of PBR in apoptosis has only been shown for immune cells (Marchetti et al, 1996a; Hirsch et al, 1998; Bono et al, 1999; Larochette et al, 1999; Ravagnan et al, 1999; Tanimoto et al, 1999) and hepatic stellate cells (Fischer et al, 2001), but little is known for cancer cells so far. To evaluate whether the observed antiproliferative effects of PBR ligands were due to induction of apoptosis caspase-3 activity was investigated.

Caspase-3 is a key-enzyme in the apoptotic signalling pathway (Nunez et al, 1998). All 3 tested PBR-specific ligands, FGIN-1-27, PK 11195 and Ro5-4864, significantly induced an increase in caspase-3 activity in HT29 cells in a dose-dependent manner. Whereas FGIN-1-27 induced a maximal activity after 6 h of incubation (Figure 4B), the maximal effect of PK 11195 and Ro5-4864 did not occur before 16 h of incubation (Figure 4C). In contrast, neither FGIN–1-52 nor clonazepam elevated caspase-3 activity. Likewise to HT29 cells, FGIN-1-27 and PK11195 dose-dependently induced caspase-3 activity in the colorectal cancer cell lines LS174T and Colo320 DM. After 16 h of incubation FGIN-1-27 increased the caspase-3 activity to 170 ± 27% (50 μM) and 204 ± 34% (100 μM) in LS174T cells and to 131 ± 1% (50 μM) and 160 ± 21% (100 μM) in Colo320 DM cells. PK11195 increased the caspase-3 activity to 113 ± 5% (50 μM) and 219 ± 1% (100 μM) in LS174T cells and to 125 ± 8% (50 μM) and 157 ± 1% (100 μM) in Colo320 DM cells. Similarly, in primary cell cultures of colorectal cancers 50 μM FGIN-1-27 elicited a significant increase of caspase-3 activity ($P < 0.01$; Figure 4C).

### Induction of cell cycle arrest in $G_1/G_0$-phase by PBR ligands

To test whether the antiproliferative effects of the PBR ligands are caused not only by an induction of apoptosis but also by an alteration of cell cycle regulation, we investigated the cell cycle by propidium iodide staining and subsequent flow cytometry analysis. The PBR ligands FGIN-1-27, PK 11195 and Ro5-4864 dose-dependently arrested HT29 cells in the $G_1/G_0$-phase of cell cycle (Figure 5) thereby decreasing the proportion of cells in the S-phase and $G_2$/M-phase. Control experiments with clonazepam or FGIN–1-52 (up to 100 μM) showed no alteration in cell cycle. In LS174T and Colo320 DM cells FGIN-1-27 and PK11195 showed similar effects. Both ligands induced an arrest in the $G_1/G_0$-phase of the cell cycle in both cell lines. FGIN-1-27 increased the proportion of cells in the $G_1/G_0$-phase from 63 ± 2% (control) to 80 ± 2% (50 μM) and 84 ± 2% (100 μM) for LS174T cells, and from 47 ± 1% (control) to 56 ± 1% (50 μM) and 61 ± 3% (100 μM) for Colo320 DM cells. Similarly, PK11195 increased the proportion of cells in the $G_1/G_0$-phase to 73 ± 1% (50 μM) and 86 ± 6% (100 μM) in LS174T cells, and to 51 ± 0.1% (50 μM) and 69 ± 5% (100 μM) in Colo320 DM cells.

### DISCUSSION

A tightly regulated balance between cell division and cell death is a prerequisite for normal tissue development. Impaired regulation of either process can lead to tumorigenesis. In this study we show that specific PBR ligands induce both an arrest of the cell cycle and an increase in apoptosis in human colorectal cancer cells. Our data indicate that PBR might be involved in both the regulation of cell division and cell death, suggesting an important role of PBR

 *© 2001 Cancer Research Campaign*

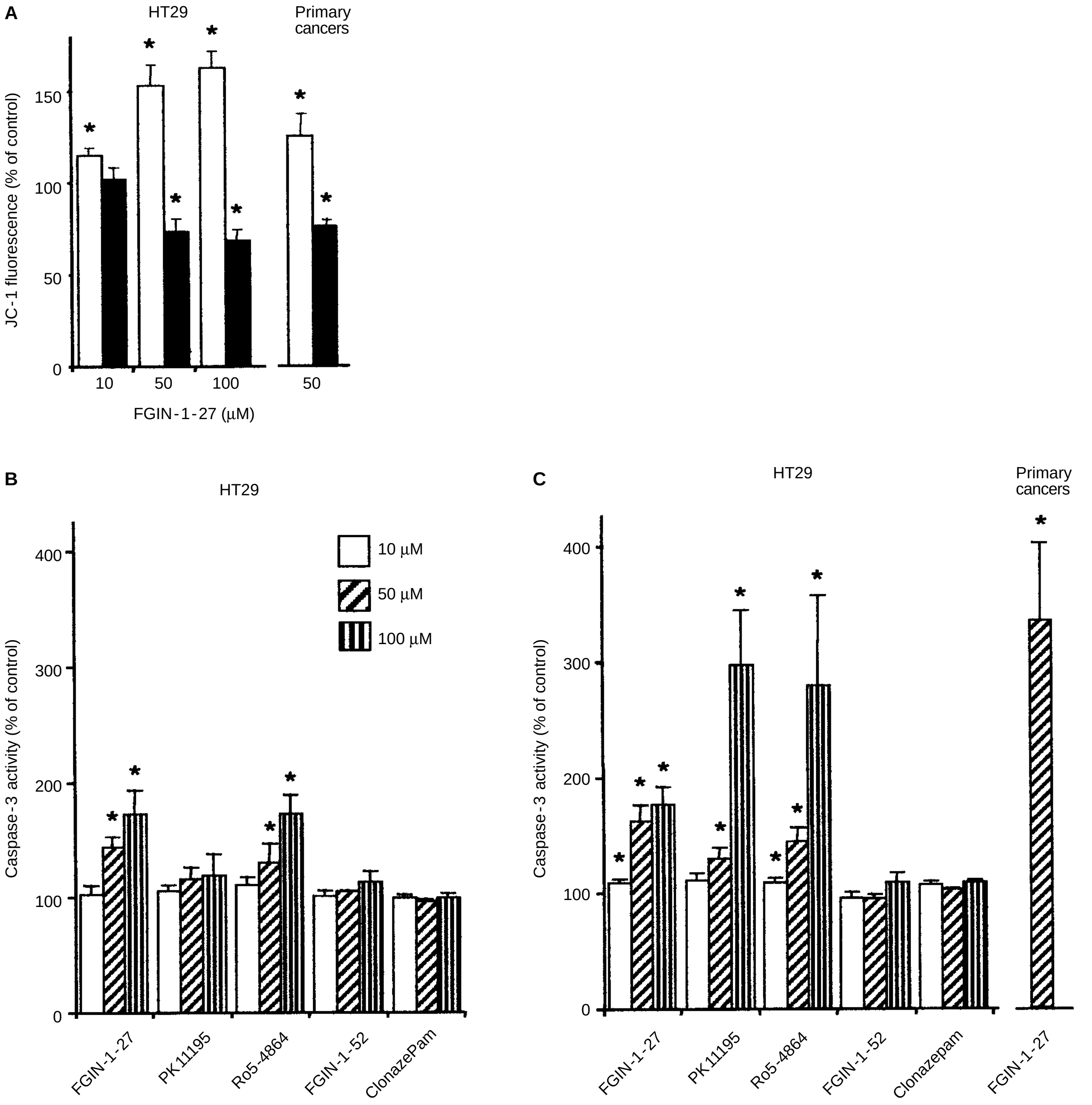

**Figure 4** Induction of mitochondrial alterations and apoptosis by PBR ligands. (**A**) After 6 h of incubation with FGIN-1-27 $\Delta\Psi_M$ (black columns) and mitochondrial volume (white columns) of HT29 cells and primary cultures of colorectal tumours were measured using the mitochondrial dye JC-1. $\Delta\Psi_M$ is indicated by mean 590 nm: 530 nm fluorescence ratio, mitochondrial volume by mean 530 nm fluorescence. Means ± SEM of 6 independent experiments with HT29 cells and of primary tumours from 6 patients are shown as percentage of control. $^*P < 0.05$ versus control without agent. (**B**, **C**) HT29 cells were incubated for 6 h (**B**) or 16 h (**C**) with 10 µM to 100 µM of FGIN-1-27, PK 11195, Ro5-4864, FGIN-1-52, or clonazepam and caspase-3 activity was determined. Means as percentage of control ± SEM of 4 independent experiments are shown. In addition, caspase-3 activity was determined of primary tumour cells of 6 patients after incubation with 50 µM FGIN-1-27. Mean ± SEM are shown. $^*P < 0.05$ versus control without agent in colorectal cancer growth. Moreover, we provide evidence that the cell cycle interfering and proapoptotic effects of specific PBR ligands are associated with an inhibition of proliferation of colorectal cancer cells.

The PBR is mainly located in the outer membranes of mitochondria (Gavish et al, 1989). Recently, a perinuclear or nuclear localization has been described in breast cancer and glioma cells, which was shown to be associated with an aggressive tumour type (Hardwick et al, 1999; Brown et al, 2000). Using confocal laser scanning microscopy and immunohistochemistry, we studied PBR expression in human colorectal cancers of distinct grades of differentiation, and in several colorectal cancer cell lines. In all 29 cancers and in the 3 cell lines studied, PBR was found to be localized in the mitochondria. Thus, PBR is primarily targeted to mitochondrial membranes of either well, moderately or poorly differentiated colorectal cancers.

To investigate the functional involvement of PBR in colorectal cancer growth we used specific exogenous ligands. These ligands were shown to interfere with the regulation of both the cell cycle and apoptosis. The specific PBR ligands induced cell cycle arrest. Upon ligand treatment, the proportion of colorectal cancer cells in the $G_1/G_0$-phase increased markedly, indicating that the cells

© 2001 Cancer Research Campaign 

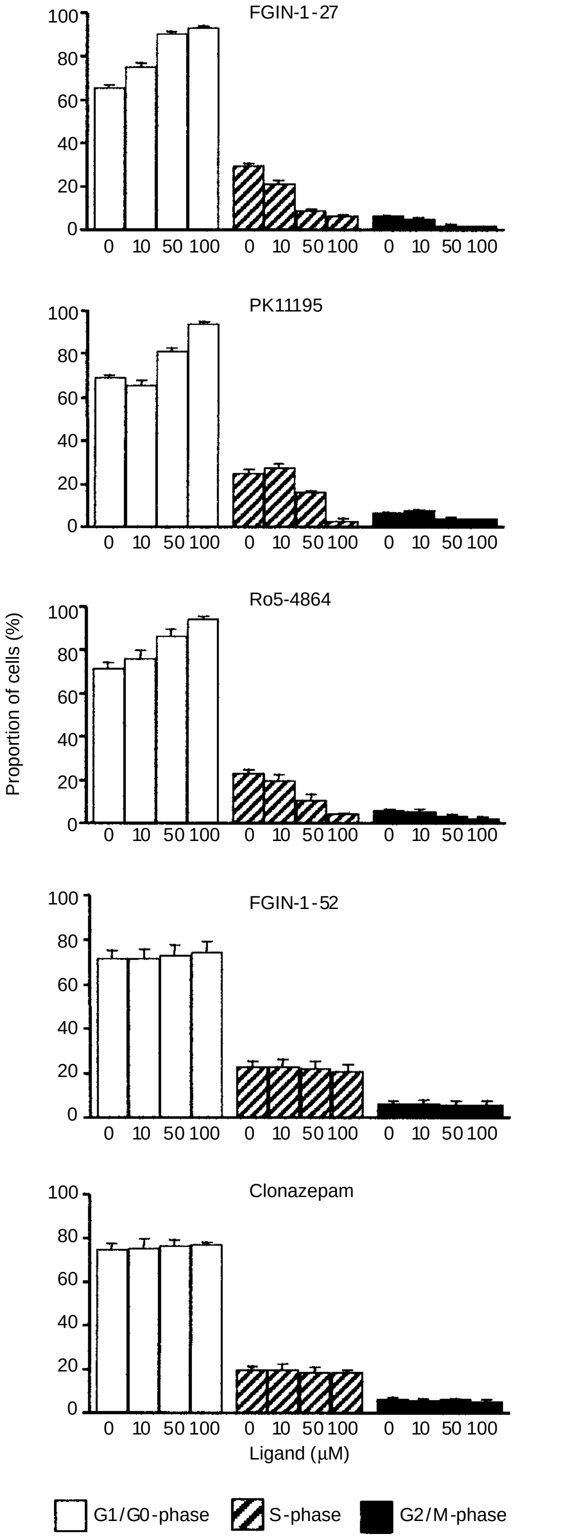

**Figure 5** Induction of cell cycle arrest by PBR ligands. After 24 h of incubation the PBR ligands FGIN-1-27, PK 11195, or Ro5-4864, dose-dependently induced an arrest in the $G_1/G_0$-phase of the cell cycle (white columns), whereas the proportion of HT29 cells in S-phase (hatched columns) and G2/M-phase (black columns) decreased. FGIN-1-52, or clonazepam did not affect the cell cycle. Means ± SEM of 3 independent experiments are shown. Difference of the proportion of cells in a respective phase of cell cycle versus control was significant for FGIN-1-27, PK 11195 and Ro5-4864 from 50 μM to 100 μM ($P < 0.05$)

stayed longer in this phase of the cell cycle. This suggests that PBR ligands act at the classical $G_1$ checkpoint, preventing cells from entering the S-phase. All 3 PBR ligands studied induced cell cycle arrest at this restriction point, suggesting a common signalling pathway. Cell cycle interfering effects of PBR ligands have been shown previously. In breast cancer PBR ligands induced a cell cycle arrest at both major restriction points, the $G_1$/S- and the $G_2$/M-junction (Carmel et al, 1999), whereas in lung and melanoma cells an accumulation in the $G_2$/M-phase was observed (Camins et al, 1995; Landau et al, 1998). These differences may reflect tissue-specific PBR signal transduction.

In addition to their cell cycle-arresting effects, we here showed for the first time that specific PBR ligands can induce apoptosis in non-haematopoietic cancer cells. The PBR ligands FGIN-1-27, PK 11195 and Ro5-4864 stimulated caspase-3 activity in the 3 cell lines and in all 6 primary cultures of colorectal cancers. Recently, mitochondrial permeability transition has been described as initiating event in the process of apoptosis (Susin et al, 1998). Within the mitochondria, PBR is known to be located in the membrane forming the permeability transition pore (Zorov, 1996). This pore is considered to play a central role in the initiation of apoptosis (Zamzami et al, 1995). In this study, the PBR ligand FGIN-1-27 decreased $\Delta\psi_M$ and caused mitochondrial swelling. Whether FGIN-1-27 induced mitochondrial alterations are a prerequisite for the induction of apoptosis in colorectal cancer cells is not yet understood. It has been reported that mitochondrial permeability transition occurs independently of the initiation of the apoptotic cascade (Lemasters et al, 1998). The actual role of the mitochondrial transition in PBR ligand-induced apoptosis needs to be further investigated.

In this study, we showed that the cell cycle-arresting and proapoptotic effects resulted in an inhibition of cell proliferation. In line with previous studies in a variety of tumour models (Garnier et al, 1993; Neary et al, 1995; Landau et al, 1998; Carmel et al, 1999; Beinlich et al, 1999), the specific PBR ligands FGIN-1-27, PK 11195 and Ro5-4864 exhibited antiproliferative effects in colorectal cancer cells at micromolar concentrations. To clarify the PBR specificity of the observed effects, we used the benzodiazepine clonazepam or the indoleacetamide FGIN–1-52. Despite their very similar structure to Ro5-4864 or FGIN-1-27, respectively, neither clonazepam nor FGIN–1-52 affected apoptosis, cell cycle or the proliferation of human colorectal cancer cells. This indicates that the effects are specific for PBR. Nevertheless, there is a quantitative discrepancy between the micromolar ligand concentrations necessary to inhibit cell proliferation and the nanomolar-binding affinities. This discrepancy might be due to the existence of a putative 'low-affinity PBR' which has previously been suggested to exist in other tissues (Pawlikowski et al, 1988; Kunert-Radek et al, 1994). This putative low-affinity binding site has been implied in the regulation of calcium channels (Cantor et al, 1984; Taft and DeLorenzo, 1984), suggesting that PBR ligands might affect calcium influx responsible for growth inhibition. However, other factors including cellular absorption, ligand metabolism, as well as differences in the conditions used for the cell proliferation and binding assays (Wang et al, 1984b) have yet to be ruled out.

Even though the exact targets and mechanisms of the proapoptotic and antiproliferative effects of the specific PBR ligands remain to be elucidated, the ability of these ligands to modulate both apoptosis and cell cycle regulation qualify them as promising agents for innovative treatment strategies in colorectal cancer disease.

 *© 2001 Cancer Research Campaign*

### ACKNOWLEDGEMENTS

We thank Britta Jebautzke for technical assistance and Dr Benno Mann for his support regarding the primary cell cultures of human colorectal cancers. We are indebted to the Institute of Physiology, Free University Berlin, Germany, for lab facilities. This study was supported by grants of the Deutsche Forschungsgemeinschaft, Deutsche Krebshilfe and the Sonnenfeld Stiftung.

### REFERENCES

Altschul SF, Madden TL, Schaffer AA, Zhang J, Zhang Z, Miller W and Lipman DJ (1997) Gapped BLAST and PSI-BLAST: a new generation of protein database search programs. *Nucleic Acids Res* **25**: 3389–3402

Anholt RR, Pedersen PL, De Souza EB and Snyder SH (1986) The peripheral-type benzodiazepine receptor. Localization to the mitochondrial outer membrane. *J Biol Chem* **261**: 576–583

Batra S and Iosif CS (1998) Elevated concentrations of mitochondrial peripheral benzodiazepine receptors in ovarian tumors. *Int J Oncol* **12**: 1295–1298

Beinlich A, Strohmeier R, Kaufmann M and Kuhl H (1999) Specific binding of benzodiazepines to human breast cancer cell lines. *Life Sci* **65**: 2099–2108

Beurdeley-Thomas A, Miccoli L, Oudard S, Dutrillaux B and Poupon MF (2000) The peripheral benzodiazepine receptors: a review. *J Neurooncol* **46**: 45–56

Bono F, Lamarche I, Prabonnaud V, Le Fur G and Herbert JM (1999) Peripheral benzodiazepine receptor agonists exhibit potent antiapoptotic activities. *Biochem Biophys Res Commun* **265**: 457–461

Braestrup C and Squires RF (1977) Specific benzodiazepine receptors in rat brain characterized by high-affinity ($^3$H)diazepam binding. *Proc Natl Acad Sci USA* **74**: 3805–3809

Brown RC, Degenhardt B, Kotoula M and Papadopoulos V (2000) Location-dependent role of the human glioma cell peripheral-type benzodiazepine receptor in proliferation and steroid biosynthesis. *Cancer Lett* **156**: 125–132

Camins A, Diez-Fernandez C, Pujadas E, Camarasa J and Escubedo E (1995) A new aspect of the antiproliferative action of peripheral-type benzodiazepine receptor ligands. *Eur J Pharmacol* **272**: 289–292

Canat X, Guillaumont A, Bouaboula M, Poinot-Chazel C, Derocq JM, Carayon P, LeFur G and Casellas P (1993) Peripheral benzodiazepine receptor modulation with phagocyte differentiation. *Biochem Pharmacol* **46**: 551–554

Cantor EH, Kenessey A, Semenuk G and Spector S (1984) Interaction of calcium channel blockers with non-neuronal benzodiazepine binding sites. *Proc Natl Acad Sci USA* **81**: 1549–1552

Carmel I, Fares FA, Leschiner S, Scherübl H, Weisinger G and Gavish M (1999) Peripheral-type benzodiazepine receptors in the regulation of proliferation of MCF-7 human breast carcinoma cell line. *Biochem Pharmacol* **58**: 273–278

Cornu P, Benavides J, Scatton B, Hauw JJ and Philippon J (1992) Increase in omega 3 (peripheral-type benzodiazepine) binding site densities in different types of human brain tumours. A quantitative autoradiography study. *Acta Neurochir* **119**: 146–152

Cossarizza A, Baccarani-Contri M, Kalashnikova G and Franceschi C (1993) A new method for the cytofluorimetric analysis of mitochondrial membrane potential using the J-aggregate forming lipophilic cation 5,5′,6,6′-tetrachloro–1,1′,3,3′-tetraethylbenzimidazolcarbocyanine iodide (JC–1). *Biochem Biophys Res Commun* **197**: 40–45

Dussossoy D, Carayon P, Feraut D, Belugou S, Combes T, Canat X, Vidal H and Casellas P (1996) Development of a monoclonal antibody to immuno-cytochemical analysis of the cellular localization of the peripheral benzodiazepine receptor. *Cytometry* **24**: 39–48

Fennell DA, Corbo M, Pallaska A and Cotter FE (2001) Bcl-2 resistant mitochondrial toxicity mediated by the isoquinoline carboxamide PK11195 involves de novo generation of reactive oxygen species. *Br J Cancer* **84**: 1397–1404

Fischer R, Schmitt M, Bode JG and Haussinger D (2001) Expression of the peripheral-type benzodiazepine receptor and apoptosis induction in hepatic stellate cells. *Gastroenterology* **120**: 1212–1226

Garnier M, Boujrad N, Oke BO, Brown AS, Riond J, Ferrara P, Shoyab M, Suarez-Quian CA and Papadopoulos V (1993) Diazepam binding inhibitor is a paracrine/autocrine regulator of Leydig cell proliferation and steroidogenesis: action via peripheral-type benzodiazepine receptor and independent mechanisms. *Endocrinology* **132**: 444–458

Gavish M, Katz Y, Bar-Ami S and Weizman R (1992) Biochemical, physiological, and pathological aspects of the peripheral benzodiazepine receptor. *J Neurochem* **58**: 1589–1601

Gavish M, Bachman I, Shoukrun R, Katz Y, Veenman L, Weisinger G and Weizman A (1999) Enigma of the peripheral benzodiazepine receptor. *Pharmacol Rev* **51**: 629–650

Gillies RJ, Didier N and Denton M (1986) Determination of cell number in monolayer cultures. *Anal Biochem* **159**: 109–113

Glassmeier G, Herzig KH, Höpfner M, Lemmer K, Jansen A and Scherübl H (1998) Expression of functional $GABA_A$ receptors in cholecystokinin-secreting gut neuroendocrine murine STC–1 cells. *J Physiol (Lond)* **510**: 805–814

Grabowski P, Mann B, Mansmann U, Lovin N, Foss HD, Berger G, Scherübl H, Riecken EO, Buhr HJ and Hanski C (2000) Expression of SIALYL-Le$^{(x)}$ antigen defined by MAb AM-3 is an independent prognostic marker in colorectal carcinoma patients. *Int J Cancer* **88**: 281–286

Grabowski P, Schindler I, Anagnostopoulos I, Foss HD, Riecken EO, Mansmann U, Stein H, Berger G, Buhr HJ and Scherübl H (2001) Neuroendocrine differentiation is a relevant prognostic factor in stage III-IV colorectal cancer. *Eur J Gastroentol Hepatol* **13**: 405–415

Hanski C, Scherübl H and Mann B (2000) Colorectal cancer: New aspects of molecular biology and immunology and their clinical applications. *Ann NY Acad Sci* **910**: 1–273

Hardwick M, Fertikh D, Culty M, Li H, Vidic B and Papadopoulos V (1999) Peripheral-type benzodiazepine receptor (PBR) in human breast cancer: correlation of breast cancer cell aggressive phenotype with PBR expression, nuclear localization, and PBR-mediated cell proliferation and nuclear transport of cholesterol. *Cancer Res* **59**: 831–842

Hirsch JD, Beyer CF, Malkowitz L, Beer B and Blume AJ (1989) Mitochondrial benzodiazepine receptors mediate inhibition of mitochondrial respiratory control. *Mol Pharmacol* **35**: 157–163

Hirsch T, Decaudin D, Susin SA, Marchetti P, Larochette N, Resche-Rigon M and Kroemer G (1998) PK11195, a ligand of the mitochondrial benzodiazepine receptor, facilitates the induction of apoptosis and reverses Bcl-2-mediated cytoprotection. *Exp Cell Res* **241**: 426–434

Höpfner M, Lemmer K, Jansen A, Hanski C, Riecken EO, Gavish M, Mann B, Buhr H, Glassmeier G and Scherübl H (1998) Expression of functional P2-purinergic receptors in primary cultures of human colorectal carcinoma cells. *Biochem Biophys Res Commun* **251**: 811–817

Ikezaki K and Black KL (1990) Stimulation of cell growth and DNA synthesis by peripheral benzodiazepine. *Cancer Lett* **49**: 115–120

Katz Y, Ben-Baruch G, Kloog Y, Menczer J and Gavish M (1990a) Increased density of peripheral benzodiazepine-binding sites in ovarian carcinomas as compared with benign ovarian tumours and normal ovaries. *Clin Sci* **78**: 155–158

Katz Y, Eitan A and Gavish M (1990b) Increase in peripheral benzodiazepine binding sites in colonic adenocarcinoma. *Oncology* **47**: 139–142

Kozikowski AP, Ma D, Brewer J, Sun S, Costa E, Romeo E and Guidotti A (1993) Chemistry, binding affinities, and behavioral properties of a new class of “antineophobic” mitochondrial DBI receptor complex (mDRC) ligands. *J Med Chem* **36**: 2908–2920

Krueger KE (1995) Molecular and functional properties of mitochondrial benzodiazepine receptors. *Biochim Biophys Acta* **1241**: 453–470

Kunert-Radek J, Stepien H and Pawlikowski M (1994) Inhibition of rat pituitary tumor cell proliferation by benzodiazepines in vitro. *Neuroendocrinology* **59**: 92–96

Laird HE, Gerrish KE, Duerson KC, Putnam CW and Russell DH (1989) Peripheral benzodiazepine binding sites in Nb 2 node lymphoma cells: effects on prolactin-stimulated proliferation and ornithine decarboxylase activity. *Eur J Pharmacol* **171**: 25–35

Landau M, Weizman A, Zoref-Shani E, Beery E, Wasseman L, Landau O, Gavish M, Brenner S and Nordenberg J (1998) Antiproliferative and differentiating effects of benzodiazepine receptor ligands on B16 melanoma cells. *Biochem Pharmacol* **56**: 1029–1034

Larochette N, Decaudin D, Jacotot E, Brenner C, Marzo I, Susin SA, Zamzami N, Xie Z, Reed J and Kroemer G (1999) Arsenite induces apoptosis via a direct effect on the mitochondrial permeability transition pore. *Exp Cell Res* **249**: 413–421

Le Fur G, Perrier ML, Vaucher N, Imbault F, Flamier A, Benavides J, Uzan A, Renault C, Dubroeucq MC and Gueremy C (1983) Peripheral benzodiazepine binding sites: effect of PK 11195, 1-(2-chlorophenyl)-N-methyl-N-(1-methylpropyl)-3-isoquinolinecarboxamide. I. In vitro studies. *Life Sci* **32**: 1839–1847

Lemasters JJ, Nieminen AL, Qian T, Trost LC, Elmore SP, Nishimura Y, Crowe RA, Cascio WE, Bradham CA, Brenner DA and Herman B (1998) The mitochondrial permeability transition in cell death: a common mechanism in necrosis, apoptosis and autophagy. *Biochim Biophys Acta* **1366**: 177–196

Maaser K, Wolf K, Klein CE, Niggemann B, Zanker KS, Brocker EB and Friedl P (1999) Functional hierarchy of simultaneously expressed adhesion receptors:

© 2001 Cancer Research Campaign integrin alpha2beta 1 but ot CD44 mediates MV3 melanoma cell migration and matrix reorganization within three-dimensional hyaluronan-containing collagen matrices. *Mol Biol Cell* **10**: 3067–3079

Mancini M, Anderson BO, Caldwell E, Sedghinasab M, Paty PB and Hockenbery DM (1997) Mitochondrial proliferation and paradoxical membrane depolarization during terminal differentiation and apoptosis in a human colon carcinoma cell line. *J Cell Biol* **138**: 449–469

Marchetti P, Hirsch T, Zamzami N, Castedo M, Decaudin D, Susin SA, Masse B and Kroemer G (1996a) Mitochondrial permeability transition triggers lymphocyte apoptosis. *J Immunol* **157**: 4830–4836

Marchetti P, Trincavelli L, Giannarelli R, Giusti L, Coppelli A, Martini C, Navalesi R and Lucacchini A (1996b) Characterization of peripheral benzodiazepine receptors in purified large mammal pancreatic islets. *Biochem Pharmacol* **51**: 1437–1442

Neary JT, Jorgensen SL, Oracion AM, Bruce JH and Norenberg MD (1995) Inhibition of growth factor-induced DNA synthesis in astrocytes by ligands of peripheral-type benzodiazepine receptors. *Brain Res* **675**: 27–30

Nicholson DW, Ali A, Thornberry NA, Vaillancourt JP, Ding CK, Gallant M, Gareau Y, Griffin PR, Labelle M and Lazebnik YA (1995) Identification and inhibition of the ICE/CED-3 protease necessary for mammalian apoptosis. *Nature* **376**: 37–43

Nunez G, Benedict MA, Hu Y and Inohara N (1998) Caspases: the proteases of the apoptotic pathway. *Oncogene* **17**: 3237–3245

Papadopoulos V (1993) Peripheral-type benzodiazepine/diazepam binding inhibitor receptor: biological role in steroidogenic cell function. *Endocr Rev* **14**: 222–240

Pawlikowski M, Kunert-Radek J, Radek A and Stepien H (1998) Inhibition of cell proliferation of human gliomas by benzodiazepines in vitro. *Acta Neurol Scand* **77**: 231–233

Petit PX, O'Connor JE, Grunwald D and Brown SC (1990) Analysis of the membrane potential of rat- and mouse-liver mitochondria by flow cytometry and possible applications. *Eur J Biochem* **194**: 389–397

Ravagnan L, Marzo I, Costantini P, Susin SA, Zamzami N, Petit PX, Hirsch F, Goulbern M, Poupon MF, Miccoli L, Xie Z, Reed JC and Kroemer G (1999) Lonidamine triggers apoptosis via a direct, Bcl-2-inhibited effect on the mitochondrial permeability transition pore. *Oncogene* **18**: 2537–2546

Stoebner PE, Carayon P, Penarier G, Frechin N, Barneon G, Casellas P, Cano JP, Meynadier J and Meunier L (1999) The expression of peripheral benzodiazepine receptors in human skin: the relationship with epidermal cell differentiation. *Br J Dermatol* **140**: 1010–1016

Susin SA, Zamzami N and Kroemer G (1998) Mitochondria as regulators of apoptosis: doubt no more. *Biochim Biophys Acta* **1366**: 151–165

Taft WC and DeLorenzo RJ (1984) Micromolar-affinity benzodiazepine receptors regulate voltage-sensitive calcium channels in nerve terminal preparations. *Proc Natl Acad Sci USA* **81**: 3118–3122

Tanimoto Y, Onishi Y, Sato Y and Kizaki H (1999) Benzodiazepine receptor agonists modulate thymocyte apoptosis through reduction of the mitochondrial transmembrane potential. *Jpn J Pharmacol* **79**: 177–183

Torres SR, Nardi GM, Ferrara P, Ribeiro-do-Valle RM and Farges RC (1999) Potential role of peripheral benzodiazepine receptors in inflammatory responses. *Eur J Pharmacol* **385**: R1–R2

Venturini I, Zeneroli ML, Corsi L, Avallone R, Farina F, Alho H, Baraldi C, Ferrarese C, Pecora N, Frigo M, Ardizzone G, Arrigo A, Pellicci R and Baraldi M (1998) Up-regulation of peripheral benzodiazepine receptor system in hepatocellular carcinoma. *Life Sci* **63**: 1269–1280

Verma A and Snyder SH (1989) Peripheral type benzodiazepine receptors. *Annu Rev Pharmacol Toxicol* **29**: 307–322

Vindelov L and Christensen IJ (1990) An integrated set of methods for routine flow cytometric DNA analysis. *Methods Cell Biol* **33**: 127–137

Wang JK, Morgan JI and Spector S (1984a) Benzodiazepines that bind at peripheral sites inhibit cell proliferation. *Proc Natl Acad Sci USA* **81**: 753–756

Wang JK, Taniguchi T and Spector S (1984b) Structural requirements for the binding of benzodiazepines to their peripheral-type sites. *Mol Pharmacol* **25**: 349–351

Xia W, Spector S, Hardy L, Zhao S, Saluk A, Alemane L and Spector NL (2000) Tumor selective G2/M cell cycle arrest and apoptosis of epithelial and hematological malignancies by BBL22, a benzazepine. *Proc Natl Acad Sci USA* **97**: 7494–7499

Zamzami N, Marchetti P, Castedo M, Decaudin D, Macho A, Hirsch T, Susin SA, Petit PX, Mignotte B and Kroemer G (1995) Sequential reduction of mitochondrial transmembrane potential and generation of reactive oxygen species in early programmed cell death. *J Exp Med* **182**: 367–377

Zorov DB (1996) Mitochondrial damage as a source of diseases and aging: a strategy of how to fight these. *Biochim Biophys Acta* **1275**: 10–15

*© 2001 Cancer Research Campaign*

## References

[PDF_00806] Altschul S. F., Madden T. L., Schäffer A. A., Zhang J., Zhang Z., Miller W., Lipman D. J. (1997). Gapped BLAST and PSI-BLAST: a new generation of protein database search programs.. Nucleic Acids Res.

[PDF_00809] Anholt R. R., Pedersen P. L., De Souza E. B., Snyder S. H. (1986). The peripheral-type benzodiazepine receptor. Localization to the mitochondrial outer membrane.. J Biol Chem.

[PDF_00812] Batra S., Iosif C. S. (1998). Elevated concentrations of mitochondrial peripheral benzodiazepine receptors in ovarian tumors.. Int J Oncol.

[PDF_00814] Beinlich A., Strohmeier R., Kaufmann M., Kuhl H. (1999). Specific binding of benzodiazepines to human breast cancer cell lines.. Life Sci.

[PDF_00816] Beurdeley-Thomas A., Miccoli L., Oudard S., Dutrillaux B., Poupon M. F. (2000). The peripheral benzodiazepine receptors: a review.. J Neurooncol.

[PDF_00818] Bono F., Lamarche I., Prabonnaud V., Le Fur G., Herbert J. M. (1999). Peripheral benzodiazepine receptor agonists exhibit potent antiapoptotic activities.. Biochem Biophys Res Commun.

[PDF_00821] Braestrup C., Squires R. F. (1977). Specific benzodiazepine receptors in rat brain characterized by high-affinity (3H)diazepam binding.. Proc Natl Acad Sci U S A.

[PDF_00825] Brown R. C., Degenhardt B., Kotoula M., Papadopoulous V. (2000). Location-dependent role of the human glioma cell peripheral-type benzodiazepine receptor in proliferation and steroid biosynthesis.. Cancer Lett.

[PDF_00828] Camins A., Diez-Fernandez C., Pujadas E., Camarasa J., Escubedo E. (1995). A new aspect of the antiproliferative action of peripheral-type benzodiazepine receptor ligands.. Eur J Pharmacol.

[PDF_00831] Canat X., Guillaumont A., Bouaboula M., Poinot-Chazel C., Derocq J. M., Carayon P., LeFur G., Casellas P. (1993). Peripheral benzodiazepine receptor modulation with phagocyte differentiation.. Biochem Pharmacol.

[PDF_00834] Cantor E. H., Kenessey A., Semenuk G., Spector S. (1984). Interaction of calcium channel blockers with non-neuronal benzodiazepine binding sites.. Proc Natl Acad Sci U S A.

[PDF_00837] Carmel I., Fares F. A., Leschiner S., Scherübl H., Weisinger G., Gavish M. (1999). Peripheral-type benzodiazepine receptors in the regulation of proliferation of MCF-7 human breast carcinoma cell line.. Biochem Pharmacol.

[PDF_00840] Cornu P., Benavides J., Scatton B., Hauw J. J., Philippon J. (1992). Increase in omega 3 (peripheral-type benzodiazepine) binding site densities in different types of human brain tumours. A quantitative autoradiography study.. Acta Neurochir (Wien).

[PDF_00844] Cossarizza A., Baccarani-Contri M., Kalashnikova G., Franceschi C. (1993). A new method for the cytofluorimetric analysis of mitochondrial membrane potential using the J-aggregate forming lipophilic cation 5,5',6,6'-tetrachloro-1,1',3,3'-tetraethylbenzimidazolcarbocyanine iodide (JC-1).. Biochem Biophys Res Commun.

[PDF_00849] Dussossoy D., Carayon P., Feraut D., Belugou S., Combes T., Canat X., Vidal H., Casellas P. (1996). Development of a monoclonal antibody to immuno-cytochemical analysis of the cellular localization of the peripheral benzodiazepine receptor.. Cytometry.

[PDF_00853] Fennell D. A., Corbo M., Pallaska A., Cotter F. E. (2001). Bcl-2 resistant mitochondrial toxicity mediated by the isoquinoline carboxamide PK11195 involves de novo generation of reactive oxygen species.. Br J Cancer.

[PDF_00857] Fischer R., Schmitt M., Bode J. G., Häussinger D. (2001). Expression of the peripheral-type benzodiazepine receptor and apoptosis induction in hepatic stellate cells.. Gastroenterology.

[PDF_00861] Garnier M., Boujrad N., Oke B. O., Brown A. S., Riond J., Ferrara P., Shoyab M., Suarez-Quian C. A., Papadopoulos V. (1993). Diazepam binding inhibitor is a paracrine/autocrine regulator of Leydig cell proliferation and steroidogenesis: action via peripheral-type benzodiazepine receptor and independent mechanisms.. Endocrinology.

[PDF_00868] Gavish M., Bachman I., Shoukrun R., Katz Y., Veenman L., Weisinger G., Weizman A. (1999). Enigma of the peripheral benzodiazepine receptor.. Pharmacol Rev.

[PDF_00865] Gavish M., Katz Y., Bar-Ami S., Weizman R. (1992). Biochemical, physiological, and pathological aspects of the peripheral benzodiazepine receptor.. J Neurochem.

[PDF_00871] Gillies R. J., Didier N., Denton M. (1986). Determination of cell number in monolayer cultures.. Anal Biochem.

[PDF_00873] Glassmeier G., Herzig K. H., Höpfner M., Lemmer K., Jansen A., Scherubl H. (1998). Expression of functional GABAA receptors in cholecystokinin-secreting gut neuroendocrine murine STC-1 cells.. J Physiol.

[PDF_00877] Grabowski P., Mann B., Mansmann U., Lövin N., Foss H. D., Berger G., Scherübl H., Riecken E. O., Buhr H. J., Hanski C. (2000). Expression of SIALYL-Le(x) antigen defined by MAb AM-3 is an independent prognostic marker in colorectal carcinoma patients.. Int J Cancer.

[PDF_00881] Grabowski P., Schindler I., Anagnostopoulos I., Foss H. D., Riecken E. O., Mansmann U., Stein H., Berger G., Buhr H. J., Scherübl H. (2001). Neuroendocrine differentiation is a relevant prognostic factor in stage III-IV colorectal cancer.. Eur J Gastroenterol Hepatol.

[PDF_00885] Hanski C., Itzkowitz S. H. (2000). Translating the knowledge of molecular alterations that occur during colon carcinogenesis into clinically relevant solutions.. Ann N Y Acad Sci.

[PDF_00888] Hardwick M., Fertikh D., Culty M., Li H., Vidic B., Papadopoulos V. (1999). Peripheral-type benzodiazepine receptor (PBR) in human breast cancer: correlation of breast cancer cell aggressive phenotype with PBR expression, nuclear localization, and PBR-mediated cell proliferation and nuclear transport of cholesterol.. Cancer Res.

[PDF_00893] Hirsch J. D., Beyer C. F., Malkowitz L., Beer B., Blume A. J. (1989). Mitochondrial benzodiazepine receptors mediate inhibition of mitochondrial respiratory control.. Mol Pharmacol.

[PDF_00896] Hirsch T., Decaudin D., Susin S. A., Marchetti P., Larochette N., Resche-Rigon M., Kroemer G. (1998). PK11195, a ligand of the mitochondrial benzodiazepine receptor, facilitates the induction of apoptosis and reverses Bcl-2-mediated cytoprotection.. Exp Cell Res.

[PDF_00900] Höpfner M., Lemmer K., Jansen A., Hanski C., Riecken E. O., Gavish M., Mann B., Buhr H., Glassmeier G., Scherübl H. (1998). Expression of functional P2-purinergic receptors in primary cultures of human colorectal carcinoma cells.. Biochem Biophys Res Commun.

[PDF_00904] Ikezaki K., Black K. L. (1990). Stimulation of cell growth and DNA synthesis by peripheral benzodiazepine.. Cancer Lett.

[PDF_00906] Katz Y., Ben-Baruch G., Kloog Y., Menczer J., Gavish M. (1990). Increased density of peripheral benzodiazepine-binding sites in ovarian carcinomas as compared with benign ovarian tumours and normal ovaries.. Clin Sci (Lond).

[PDF_00909] Katz Y., Eitan A., Gavish M. (1990). Increase in peripheral benzodiazepine binding sites in colonic adenocarcinoma.. Oncology.

[PDF_00911] Kozikowski A. P., Ma D., Brewer J., Sun S., Costa E., Romeo E., Guidotti A. (1993). Chemistry, binding affinities, and behavioral properties of a new class of "antineophobic" mitochondrial DBI receptor complex (mDRC) ligands.. J Med Chem.

[PDF_00915] Krueger K. E. (1995). Molecular and functional properties of mitochondrial benzodiazepine receptors.. Biochim Biophys Acta.

[PDF_00917] Kunert-Radek J., Stepień H., Pawlikowski M. (1994). Inhibition of rat pituitary tumor cell proliferation by benzodiazepines in vitro.. Neuroendocrinology.

[PDF_00920] Laird H. E., Gerrish K. E., Duerson K. C., Putnam C. W., Russell D. H. (1989). Peripheral benzodiazepine binding sites in Nb 2 node lymphoma cells: effects on prolactin-stimulated proliferation and ornithine decarboxylase activity.. Eur J Pharmacol.

[PDF_00924] Landau M., Weizman A., Zoref-Shani E., Beery E., Wasseman L., Landau O., Gavish M., Brenner S., Nordenberg J. (1998). Antiproliferative and differentiating effects of benzodiazepine receptor ligands on B16 melanoma cells.. Biochem Pharmacol.

[PDF_00928] Larochette N., Decaudin D., Jacotot E., Brenner C., Marzo I., Susin S. A., Zamzami N., Xie Z., Reed J., Kroemer G. (1999). Arsenite induces apoptosis via a direct effect on the mitochondrial permeability transition pore.. Exp Cell Res.

[PDF_00932] Le Fur G., Perrier M. L., Vaucher N., Imbault F., Flamier A., Benavides J., Uzan A., Renault C., Dubroeucq M. C., Guérémy C. (1983). Peripheral benzodiazepine binding sites: effect of PK 11195, 1-(2-chlorophenyl)-N-methyl-N-(1-methylpropyl)-3-isoquinolinecarboxamide. I. In vitro studies.. Life Sci.

[PDF_00937] Lemasters J. J., Nieminen A. L., Qian T., Trost L. C., Elmore S. P., Nishimura Y., Crowe R. A., Cascio W. E., Bradham C. A., Brenner D. A. (1998). The mitochondrial permeability transition in cell death: a common mechanism in necrosis, apoptosis and autophagy.. Biochim Biophys Acta.

[PDF_00950] Mancini M., Anderson B. O., Caldwell E., Sedghinasab M., Paty P. B., Hockenbery D. M. (1997). Mitochondrial proliferation and paradoxical membrane depolarization during terminal differentiation and apoptosis in a human colon carcinoma cell line.. J Cell Biol.

[PDF_00954] Marchetti P., Hirsch T., Zamzami N., Castedo M., Decaudin D., Susin S. A., Masse B., Kroemer G. (1996). Mitochondrial permeability transition triggers lymphocyte apoptosis.. J Immunol.

[PDF_00957] Marchetti P., Trincavelli L., Giannarelli R., Giusti L., Coppelli A., Martini C., Navalesi R., Lucacchini A. (1996). Characterization of peripheral benzodiazepine receptors in purified large mammal pancreatic islets.. Biochem Pharmacol.

[PDF_00961] Neary J. T., Jorgensen S. L., Oracion A. M., Bruce J. H., Norenberg M. D. (1995). Inhibition of growth factor-induced DNA synthesis in astrocytes by ligands of peripheral-type benzodiazepine receptors.. Brain Res.

[PDF_00964] Nicholson D. W., Ali A., Thornberry N. A., Vaillancourt J. P., Ding C. K., Gallant M., Gareau Y., Griffin P. R., Labelle M., Lazebnik Y. A. (1995). Identification and inhibition of the ICE/CED-3 protease necessary for mammalian apoptosis.. Nature.

[PDF_00967] Nuñez G., Benedict M. A., Hu Y., Inohara N. (1998). Caspases: the proteases of the apoptotic pathway.. Oncogene.

[PDF_00969] Papadopoulos V. (1993). Peripheral-type benzodiazepine/diazepam binding inhibitor receptor: biological role in steroidogenic cell function.. Endocr Rev.

[PDF_00971] Pawlikowski M., Kunert-Radek J., Radek A., Stepien H. (1988). Inhibition of cell proliferation of human gliomas by benzodiazepines in vitro.. Acta Neurol Scand.

[PDF_00974] Petit P. X., O'Connor J. E., Grunwald D., Brown S. C. (1990). Analysis of the membrane potential of rat- and mouse-liver mitochondria by flow cytometry and possible applications.. Eur J Biochem.

[PDF_00977] Ravagnan L., Marzo I., Costantini P., Susin S. A., Zamzami N., Petit P. X., Hirsch F., Goulbern M., Poupon M. F., Miccoli L. (1999). Lonidamine triggers apoptosis via a direct, Bcl-2-inhibited effect on the mitochondrial permeability transition pore.. Oncogene.

[PDF_00981] Stoebner P. E., Carayon P., Penarier G., Fréchin N., Barnéon G., Casellas P., Cano J. P., Meynadier J., Meunier L. (1999). The expression of peripheral benzodiazepine receptors in human skin: the relationship with epidermal cell differentiation.. Br J Dermatol.

[PDF_00985] Susin S. A., Zamzami N., Kroemer G. (1998). Mitochondria as regulators of apoptosis: doubt no more.. Biochim Biophys Acta.

[PDF_00987] Taft W. C., DeLorenzo R. J. (1984). Micromolar-affinity benzodiazepine receptors regulate voltage-sensitive calcium channels in nerve terminal preparations.. Proc Natl Acad Sci U S A.

[PDF_00990] Tanimoto Y., Onishi Y., Sato Y., Kizaki H. (1999). Benzodiazepine receptor agonists modulate thymocyte apoptosis through reduction of the mitochondrial transmembrane potential.. Jpn J Pharmacol.

[PDF_00996] Venturini I., Zeneroli M. L., Corsi L., Avallone R., Farina F., Alho H., Baraldi C., Ferrarese C., Pecora N., Frigo M. (1998). Up-regulation of peripheral benzodiazepine receptor system in hepatocellular carcinoma.. Life Sci.

[PDF_01000] Verma A., Snyder S. H. (1989). Peripheral type benzodiazepine receptors.. Annu Rev Pharmacol Toxicol.

[PDF_01002] Vindeløv L., Christensen I. J. (1990). An integrated set of methods for routine flow cytometric DNA analysis.. Methods Cell Biol.

[PDF_01004] Wang J. K., Morgan J. I., Spector S. (1984). Benzodiazepines that bind at peripheral sites inhibit cell proliferation.. Proc Natl Acad Sci U S A.

[PDF_01006] Wang J. K., Taniguchi T., Spector S. (1984). Structural requirements for the binding of benzodiazepines to their peripheral-type sites.. Mol Pharmacol.

[PDF_01009] Xia W., Spector S., Hardy L., Zhao S., Saluk A., Alemane L., Spector N. L. (2000). Tumor selective G2/M cell cycle arrest and apoptosis of epithelial and hematological malignancies by BBL22, a benzazepine.. Proc Natl Acad Sci U S A.

[PDF_01013] Zamzami N., Marchetti P., Castedo M., Decaudin D., Macho A., Hirsch T., Susin S. A., Petit P. X., Mignotte B., Kroemer G. (1995). Sequential reduction of mitochondrial transmembrane potential and generation of reactive oxygen species in early programmed cell death.. J Exp Med.

[PDF_01017] Zorov D. B. (1996). Mitochondrial damage as a source of diseases and aging: a strategy of how to fight these.. Biochim Biophys Acta.

